# Imaging the Proangiogenic Effects of Cardiovascular Drugs in a Diabetic Model of Limb Ischemia

**DOI:** 10.1155/2019/2538909

**Published:** 2019-02-03

**Authors:** J. L. Goggi, A. Haslop, R. Boominathan, K. Chan, V. Soh, P. Cheng, E. G. Robins, K. K. Bhakoo

**Affiliations:** ^1^Singapore Bioimaging Consortium, Agency for Science, Technology and Research (A∗ STAR), 11 Biopolis Way, #07-10 Helios, Singapore 138667; ^2^Department of Physiology, Yong Loo Lin School of Medicine, National University of Singapore, Singapore 117456; ^3^Clinical Imaging Research Centre (A∗ STAR–NUS), Yong Loo Lin School of Medicine, National University of Singapore, Singapore 117599

## Abstract

**Purpose:**

Peripheral artery disease (PAD) causes narrowing of arteries in the limbs, leading to tissue ischemia, gangrene, and eventually limb amputation. The presence of diabetes greatly exacerbates the course of PAD, accounting for the majority of lower limb amputations. Therapeutic strategies focussing on macrovascular repair are less effective in diabetic patients where smaller vessels are affected, and proangiogenic therapies offer a viable adjunct to improve vascularisation in these at risk individuals. The purpose of the current study was to assess the proangiogenic effects of drugs routinely used to treat cardiovascular disease in a diabetic murine model of hind limb ischemia longitudinally using multimodal imaging.

**Procedures:**

Diabetic mice underwent surgical intervention to induce hind limb ischemia and were treated with simvastatin, metformin, or a combination orally for 28 days and compared to diabetic and nondiabetic mice. Neovascularisation was assessed using [^18^F]FtRGD PET imaging, and macrovascular volume was assessed by quantitative time of flight MRI. At each imaging time point, VEGF expression and capillary vessel density were quantified using immunohistochemical analysis, and functional recovery and disease progression were assessed.

**Results:**

Combined use of simvastatin and metformin significantly increased neovascularisation above levels measured with either treatment alone. Early angiogenic events were accurately assessed using PET [^18^F]FtRGD, showing maximal retention in the ischemic hind limb by day 8, which translated to a sustained increase in vascular volume at later time points. Immunohistochemical analysis shows that combined therapy significantly increased VEGF expression and capillary density (CD31^+^) in a similar time course and also slowed disease progression while simultaneously improving functional foot use.

**Conclusions:**

Combined treatment with simvastatin and metformin led to a significant improvement in limb angiogenesis, vascular volume, and sustained functional recovery in a diabetic murine model of HLI. PET imaging with [^18^F]FtRGD provides a robust method for early detection of these proangiogenic effects preclinically and may be useful for the assessment of proangiogenic therapies used clinically to treat diabetic PAD patients.

## 1. Introduction

Peripheral artery disease (PAD) results from systemic atherosclerosis, causing narrowing of arteries in the limbs, leading to tissue ischemia, gangrene, and eventually limb amputation. The presence of diabetes greatly increases the risk of PAD, accounting for the majority of lower limb amputations [1]. Currently, surgical treatments that bypass the affected vessel using autologous arteries and veins or catheter-based endovascular revascularisation are feasible for larger-sized vessels (e.g., the iliac and femoral arteries); however, outcomes from these treatments in small arteries are very poor [2]. Unfortunately, patients with diabetes are much more likely to be affected by small vessel rather than large vessel obstruction leaving them with few treatment options. The use of proangiogenic therapeutics to induce the growth of blood vessels in ischemic tissues of diabetic patients has been intensively studied as an adjunct to endovascular revascularisation, but accurate measurement of the proangiogenic effect is stymied by a lack of sensitive measurement techniques. Clinically measurement of the ankle brachial index (ABI) or MR angiography is used, but these techniques are optimised for larger vessels and provide little information about angiogenesis [3]. New blood vessels have been shown to express high levels of integrins. The integrins *α*_V_ *β*_3_ and *α*_V_ *β*_5_ are expressed at low levels on epithelial cells and mature endothelial cells but are highly expressed on activated endothelial cells during neovasculature [4]. These integrins act as receptors for proteins expressing the arginine-glycine-aspartate (RGD) tripeptide motif [5]. Imaging techniques such as positron emission tomography (PET) with RGD peptide radiopharmaceuticals that target integrins have been shown to be a sensitive measure of new blood vessel growth preclinically [6, 7].

Growth factors, in particular, vascular endothelial growth factor (VEGF) delivered via recombinant proteins, viral vectors, or cells have shown promise in preclinical studies and small-scale clinical trials but met with limited success in large-scale clinical trials, with protein instability and inefficiency of delivery systems suggested as causes of failure [4, 8]. Alternative proangiogenic therapies are being investigated and a number of existing clinically approved drugs have been implicated as potential proangiogenic therapies. Two routine therapies used in cardiovascular disease have been shown to significantly increase angiogenesis in preclinical models of ischemia and could be rapidly adopted for use in PAD. Metformin, a widely used antidiabetic drug for type 2 diabetes, and simvastatin, a 3-hydroxy-3-methyl-glutaryl-CoA (HMG-CoA) reductase inhibitor, used to regulate cholesterol levels, have previously been shown to enhance recovery from acute limb ischemia in nondiabetic animals [7, 9–11], and pretreatment with simvastatin has been shown to ameliorate limb ischemia in diabetic mice [12]. We investigate whether multimodal imaging can reliably measure the subtle changes in vascular remodeling in the diabetic limb induced by treatment with metformin or simvastatin and whether combined use amplifies their therapeutic potential.

## 2. Materials and Methods

Male BALB/c mice were purchased from In Vivos (Singapore). BALB/c mice were chosen as a model animal, as they have been shown to develop the most severe initial ischemia and display relatively poor spontaneous recovery after arterial occlusion.

All chemicals and solvents (including anhydrous solvents) obtained commercially were of analytical grade and used directly without further purification.

### 2.1. Preparation of [^18^F]FtRGD (2-[(18)F]Fluoroethyl-triazolyl Conjugated *c*(RGDyK) Peptide)

2-[^18^F]fluoroethyl azide was prepared as described by Glaser and Årstad [13]. Briefly, aqueous [^18^F]fluoride (approx. 3 mL) was trapped on a preconditioned QMA carbonate cartridge (Waters, Milford, MA, USA) and eluted from the cartridge with a solution of Kryptofix K222 (5 mg, 13.3 *µ*mol) and K_2_CO_3_ (1 mg, 7.2 *µ*mol) in 150 *µ*L dry acetonitrile and 850 *µ*L water, into a 5 mL Wheaton vial. The vented vessel was heated to 130°C under N_2_ flow and K(K222)[^18^F] complex dried through azeotropic distillation. After drying, a solution of 2-azidoethyl-4-toluenesulfonate (2 *µ*L) in dry acetonitrile (0.5 mL) was added to the reaction vessel, and the mixture was heated to 90°C for 10 min to afford 2-[^18^F]fluoroethyl azide. The conjugation of 2-[^18^F]fluoroethyl azide with alkyne-functionalized *c*(RGDyK) peptide via the Cu(I)-catalysed Huisgen 1,3-dipolar cycloaddition reaction was carried out using a manual procedure adapted from the method described by Bejot et al. [14] with minor modifications. Prior to radiolabelling, a 5 mL Wheaton vial containing pentynoic *c*(RGDyK) (3 mg, 4.84 *µ*mol), gentistic acid (4 mg, 26 *µ*mol) predissolved in 1 M K_2_CO_3_ solution (100 *µ*L, 100 *µ*mol), 0.1 M bathophenanthrolinedisulfonate (BPDS) solution (16 *µ*L, 1.6 *µ*mol), and 1 M sodium ascorbate solution (12 *µ*L, 12 *µ*mol) was prepared. The mixture was purged gently with a N_2_ stream for 5 minutes before addition of 0.1 M copper sulphate solution (16 *µ*L, 1.6 *µ*mol). 2-[^18^F]Fluoroethyl azide was subsequently distilled into the mixture at 130°C under a gentle N_2_ flow. The flow was stopped, and the reaction mixture was allowed to react at 60°C for 5 minutes, after which a stream of N_2_ was used to purge the reaction mixture for a further 5 minutes to remove unreacted 2-[^18^F]fluoroethyl azide and acetonitrile. After cooling, the reaction mixture was quenched with TFA in water (0.1% v/v, 2.5 mL). [^18^F]FtRGD was isolated by semipreparative HPLC (Phenomenex Luna 5*μ* C18 100A (250 × 10 mm) column; Eluant A: solution of trifluoroacetic acid in water (0.1% v/v); Eluant B: solution of trifluoroacetic acid in acetonitrile (0.1% v/v); gradient method = 10–90% (B) in 15 min; flow rate = 3 mL/min; *λ* = 254 nm). The retention time of [^18^F]FtRGD was 6 min. The HPLC fraction containing [^18^F]FtRGD was diluted with water (15 mL), and the product was trapped on a solid-phase C_18_ light cartridge before being eluted from the cartridge with absolute ethanol (0.5 mL) and reformulated by addition of phosphate buffered saline (pH = 7.4, 4.5 mL). [^18^F]FtRGD was identified and quantified by QC HPLC (Phenomenex Luna 5*μ* C18 100A (250 × 4.6 mm) column; Eluant A: solution of trifluoroacetic acid in water (0.1% v/v); Eluant B: acetonitrile; gradient method = 10–90% (B) in 15 min; flow rate = 1 mL/min). The retention time of [^18^F]FtRGD was 9 min, the nondecay corrected radiochemical yield was 9.36 ± 1.11% (*n*=12) with a radiochemical purity of ≥99% and a molar activity of 1–3 GBq/*μ*mol in a total reaction time of 90 minutes from [^18^F]fluoride.

### 2.2. Induction of Hyperglycemia

The mice were dosed with streptozotocin (STZ, single dose at 170 mg/kg in 50 mM sodium citrate buffer pH 4.5 via intraperitoneal injection (IP)) to induce persistent hyperglycemia. After two weeks, fasting blood glucose levels were assessed to evaluate persistent hyperglycemia and determine which animals would proceed for surgery. Subsequently, fasting blood glucose was longitudinally assessed on each imaging day using a One Touch Basic blood glucose monitoring system (Lifescan, USA; animals were considered to be hyperglycemic above 7.4 mmol/liter).

### 2.3. Hind Limb Ischemia Surgery

BALB/c mice were anaesthetised using ketamine (150 mg/kg) and xylazine (10 mg/kg, IP) and the femoral artery was ligated as previously described [7]. Briefly, the external iliac artery was ligated above the pudendoepigastric trunk proximal to the deep femoral artery twice and dissected between the two ligations. The incision was closed, and the animals received subcutaneous injection of enrofloxacin (10 mg/kg, once daily) for 5 days and buprenorphine (0.1 mg/kg, twice daily) for 3 days postsurgery.

### 2.4. Laser Doppler Perfusion Imaging

**Figure 1:**
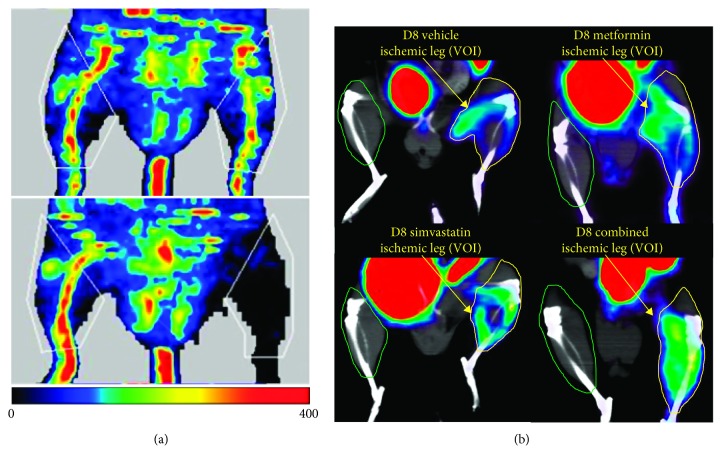
Representative images displaying (a) laser Doppler perfusion imaging assessment of tissue perfusion before (upper panel) and after femoral artery ligation (lower panel). The region of interest placement is indicated in white. (b) Representative images showing uptake of [^18^F]FtRGD in the ischemic leg at day 8 in each of the diabetic treatment groups (representative nondiabetic image not shown). The region of interest placement for the ischemic leg is indicated in yellow, and region of interest placement for the contralateral intact leg is shown in green.

The mice were imaged on the day prior to surgery and day 1 after surgery as previously described [15]. Briefly, animals were anaesthetised using isoflurane via a nose cone and maintained at 37°C and imaged in both the prone and supine position to assess perfusion of the lower limbs as shown in Figure 1(a). Regions of interest over the musculature of the lower limbs were used to assess perfusion and femoral ligation surgery success. Animals with greater than 95% reduction in perfusion in the affected limb were randomised into treatment groups for assessment.

### 2.5. Dosing

Simvastatin (2.0 mg/kg in saline, S1792 Selleckchem, USA), metformin (350 mg/kg in saline, Sigma-Aldrich USA), combined treatment (simvastatin 2 mg/kg and metformin 350 mg/kg), or vehicle (saline alone) was administered immediately following laser Doppler perfusion imaging and subsequently dosed daily (following the clinical regimen) by oral gavage for 28 days.

### 2.6. PET Imaging with [^18^F]FtRGD

The animals were repeat imaged on day 1, 3, 8, 14, 21, and 28 after surgery. The animals were injected with a solution of [^18^F]FtRGD (∼30 MBq in 0.2 ml) via the lateral tail vein, and static images were acquired from 70 to 90 min postinjection based on prior dynamic imaging studies to optimise SNR as previously described [14]. Low-dose CT images (40 kV, 500 *µ*A; 2 × 2 binning, 100 *µ*m resolution) were acquired for anatomical information. The PET and CT images were coregistered to confirm anatomical location of the [^18^F]FtRGD uptake. Uptake of radioactivity was determined by placement of a region of interest (ROI) over the muscles of interest on both the ischemic limb and the intact control limb delineated using the CT images. The muscles of interest used for ROI placement included the major musculature of the thigh and calf including the gastrocnemius, gracilis, and adductors as shown in Figure 1(b). The tissue concentrations were measured using ROI analysis in Amide software (Sourceforge 10.3, http://amide.sourceforge.net) and are presented as percent injected dose/gram (%ID/g).

### 2.7. MRI TOF Imaging

The animals were also imaged on days 1, 3, 8, 14, 21, and 28 after surgery with MRI time of flight imaging as previously described [7]. TOF-MRI was performed using a Bruker 9.4T Biospec MRI scanner using an optimised flow compensated gradient-echo time of flight (TOF) protocol with a spatial resolution of 0.109 mm/pixel × 0.109 mm/pixel, 0.35 mm slice thickness, and 150 slices and analysed using FIJI (LOCI MA, WA, USA). A background threshold was applied, and the images were analysed for pixel intensity to provide a quantitative measurement of the signal within the region of interest.

### 2.8. Functional Impairment Assessment

**Figure 2:**
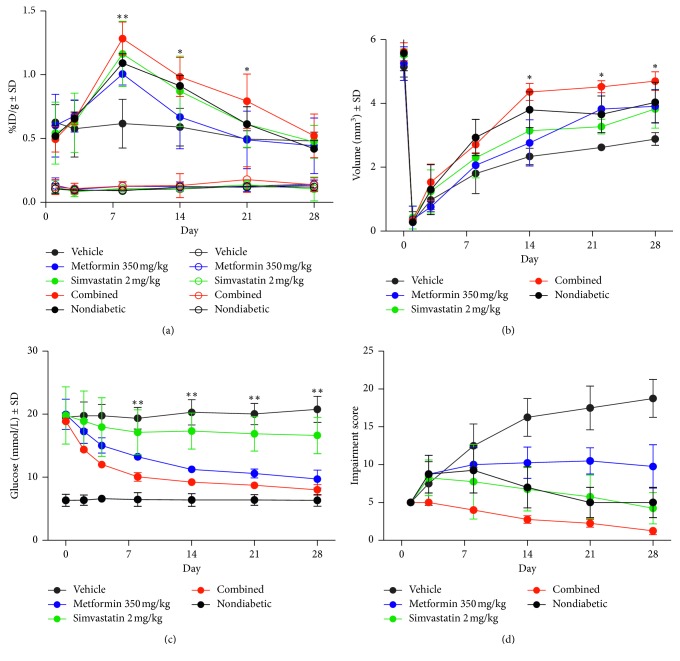
(a) Retention of [^18^F]FtRGD measured by longitudinal PET imaging (∼10 MBq, acquired from 70–90 min postinjection under isoflurane anaesthesia). Closed circles represent data from the ischemic leg and open circles represent data from the contralateral intact leg. Retention was significantly higher in the combination-treated ischemic limb from day 8 to day 28 postligation (whereas the nondiabetic ischemic limb and simvastatin-treated ischemic limb were only significantly higher on days 8 and 14 and metformin on day 8 only) compared to the vehicle-treated ischemic limb (*n*=6, ^*∗*^ *p* < 0.05, ^*∗∗*^ *p* < 0.01, 1-way ANOVA with post hoc Tukey's test, data shown as %ID/g ± SD). (b) Hind limb vascular volume measured by TOF MRI. Vascular volume was significantly higher in the nondiabetic ischemic limb, combined treatment ischemic limb, simvastatin-treated ischemic limb, and metformin-treated ischemic limb from day 21 to 28 postligation compared to the vehicle-treated ischemic limb (*n*=6, ^*∗*^ *p* < 0.05, 1-way ANOVA with post hoc Tukey's test, mean volume in mm^3^ ± SD). (c) Blood glucose concentration measured by venous blood sampling. Both metformin and combined treatment decreased the blood glucose levels from day 2 (*n*=6, ^*∗*^ *p* < 0.05, ^*∗∗*^ *p* < 0.01, 1-way ANOVA with post hoc Tukey's test, mean % area stained ± SD). (d) Observational measures of disease progression and functional limb use. Functional foot use was severely impaired in all animals early in the study with gradual improvement observed in the treated and nondiabetic groups in comparison to the vehicle-treated diabetic group. Development of progressive necrosis was observed in the vehicle-treated group; a slower progression was observed in the simvastatin-treated group, metformin-treated group, and nondiabetic group, whereas no necrosis was observed in the combined treatment group (*n*=6, ^*∗*^ *p* < 0.05, ^*∗∗*^ *p* < 0.01, 1-way ANOVA with post hoc Tukey's test, mean impairment score ± SD).

Disease progression and functional recovery after induction of ischemia were assessed by observational measurement of toe and foot necrosis and subjective use of the affected limb during walking and climbing as outlined in Table 1. Briefly, the animals were visually assessed for the development of necrosis in the affected limb over the course of the study, and animals were assessed for their ability to functionally use the affected limb for ambulatory and climbing purposes; the assessment criteria for limb damage and functional use were used for assessment at each time point. An impairment score was generated for each animal by multiplying the limb damage score by the functional use score, and the results are shown in Figure 2(d).

### 2.9. Immunohistochemical Assessment of Capillary Vessel Number and VEGF Expression

The major thighs and calf muscles (including the gracilis, abductors, adductors, gastrocnemius, and soleus) were excised immediately following each imaging time point and were fixed in neutral buffered formalin. Sections were taken throughout the muscle samples at 12 *μ*m thickness and stained for CD31 using anti-CD31 polyclonal antibody (Abcam Singapore Pte., Ltd.) or for VEGF using anti-VEGF monoclonal antibody (Abcam Singapore Pte., Ltd.). Muscle capillary number was calculated as CD31-positive vessel number and VEGF levels as VEGF-positive cells per 10 fields of view assessed per sample. Binding was analysed manually as previously described [7] in a manner blinded to treatment assignment.

## 3. Results

### 3.1. Quantification of Limb Retention of [^18^F]FtRGD Peptide

Small animal PET imaging was performed on days 1, 3, 8, 14, 21, and 28 days postinitiation of hind limb ischemia. Drug treatment was initiated on the day of surgery.  Figure 2(a) and Table S1 describe the uptake of [^18^F]FtRGD in the intact and ischemic legs over the time course assessed. On day 1 after surgery, retention of [^18^F]FtRGD in the ischemic limb was significantly reduced in all groups compared to the intact contralateral leg (*p* < 0.001, 1-way ANOVA with post hoc Tukey's test), but no significant difference in ischemic limb uptake was observed in any of the treatment groups compared to diabetic vehicle controls or nondiabetic controls. Again, no significant difference in retention of [^18^F]FtRGD was observed in the treated groups compared to diabetic vehicle controls on day 3 postsurgery. However, by day 8, [^18^F]FtRGD retention was significantly increased in the metformin-treated group, simvastatin-treated group, combination therapy group, and nondiabetic group compared to diabetic vehicle controls (*p* < 0.001). The combination-treated group showed the greatest retention in the ischemic leg at day 8 but was only marginally higher than the metformin-treated group, simvastatin-treated group, and nondiabetic group. By day 14, however, this significant increase in limb retention of [^18^F]FtRGD was only observed in the combination-treated group (*p* < 0.05) and no longer in the metformin-treated group, simvastatin-treated group, and nondiabetic group compared to diabetic vehicle controls. This increased limb retention of [^18^F]FtRGD remained significantly higher in the combination-treated group at day 21 (*p* < 0.05). By day 28, none of the groups displayed significantly different uptake of [^18^F]FtRGD. No difference in retention of [^18^F]FtRGD was observed between any group and vehicle controls in the intact contralateral legs at any of the time points.

### 3.2. Assessment of Vascular Volume Using MRI Time of Flight Imaging

MRI time of flight (TOF) imaging was performed on days 1, 3, 8, 14, 21, and 28 postinitiation of hind limb ischemia. Drug treatment was initiated on the day of surgery.  Figure 2(b) shows the vascular volume in the ischemic legs over these 28 days. The vascular volume in the contralateral intact legs can be found in Table S2; no significant difference in vascular volume was observed between any of the groups in the intact legs at any of the time points studied. The MRI TOF data show that the vascular volume in the right ischemic leg was similar in all groups prior to surgery. After surgical induction of HLI, the vascular volume in the ischemic limb was significantly reduced in all groups compared to the contralateral intact leg (*p* < 0.001, 1-way ANOVA with post hoc Tukey's test), but no significant difference in ischemic limb uptake was observed in any of the treatment groups compared to diabetic vehicle controls or nondiabetic controls. The vascular volume trended towards an increase in all treatment groups at day 3 and day 8 compared to the diabetic vehicle control but failed to reach significance. Statistically significant increases in volume were evident in the combination-treated group and the nondiabetic group only at day 14 (*p* < 0.05). At day 21 and day 28, a statistically significant improvement in *s* = vascular volume was observed in all groups in comparison to diabetic vehicle control ischemic limb values (*p* < 0.05).

### 3.3. Functional Recovery

Figure 2(d) shows the functional recovery of the ischemic limb assessed over 28 days using the criteria described in Table 1. The data show that necrosis was observed in one toenail or more in all groups except the combination-treated group, from day 1 onwards. A progression in necrosis was observed in the vehicle-treated group but was not observed to progress in any other group. Functional foot use was severely impaired in all animals by day 1 and did not improve in the vehicle-treated group over the time course studied. An observed improvement in limb use was observed in all the treated and nondiabetic groups with normal ambulatory function recovered in the majority of animals in the simvastatin, combination, and nondiabetic groups by day 21.

### 3.4. Muscle Tissue Capillary Vessel Number Analysis

**Figure 3:**
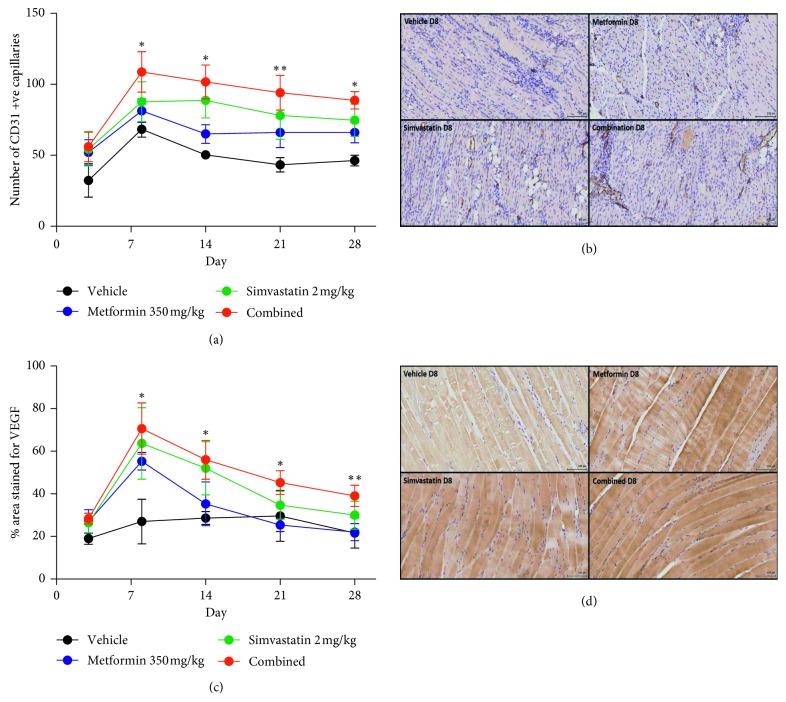
(a) Graph showing the number of CD31 positive vessels in the diabetic ischemic hind limb only measured by immunohistochemical assessment of anti-CD31 antibody staining. The number of CD31 positive capillaries was significantly higher in the combined treatment ischemic limb, simvastatin-treated ischemic limb, and metformin-treated ischemic limb from day 8 to day 28 postligation compared to the vehicle-treated ischemic limb (*n*=4, ^*∗*^ *p* < 0.05, ^*∗∗*^ *p* < 0.01 1-way ANOVA with post hoc Tukey's test, mean number of capillaries ± SD). (b) Representative muscle sections taken at day 8 displaying staining for CD31 in the diabetic vehicle-treated ischemic muscle, metformin-treated ischemic muscle, simvastatin-treated ischemic muscle, and combination-treated (D) ischemic muscle (representative nondiabetic image not shown). (c) Graph showing the percentage area of VEGF intense positive staining in diabetic hind limb muscle measured by immunohistochemical assessment of anti-VEGF antibody staining. VEGF staining was significantly higher in the combination-treated ischemic limb from day 8 to day 28 postligation (whereas simvastatin was only significantly higher on days 8 and 14 and metformin on day 8 only) compared to the vehicle-treated ischemic limb (*n*=4, ^*∗*^ *p* < 0.05, ^*∗∗*^ *p* < 0.01, 1-way ANOVA with post hoc Tukey's test, mean % area stained ± SD). (d) Representative muscle sections taken at day 8 displaying staining for VEGF in the diabetic vehicle-treated ischemic muscle, metformin-treated ischemic muscle, simvastatin-treated ischemic muscle, and combination-treated ischemic muscle (representative nondiabetic image not shown).

Muscle tissue was assessed for CD31 positively stained vessels *ex vivo* on each of the imaging days (3–28) in a separate cohort of animals (*n*=4).  Figure 3(a) and Table S3 show the number of vessels in the muscle from the ischemic and intact limbs (only shown in Table S3) on each day studied, whilst Figure 3(b) shows a representative field of view from ischemic muscle tissues on day 8. The number of vessels in the ischemic legs of the treated groups was comparable with vehicle on day 3 after surgical intervention. The number of CD31 positive vessels increased in all treated groups compared to the vehicle by day 8, but the capillary number was significantly higher in the combination-treated group (*p* < 0.05, 1-way ANOVA with post hoc Tukey's test). On the remaining days, the capillary number was significantly greater in the simvastatin (*p* < 0.05) and combination (*p* < 0.05) groups only.

### 3.5. Muscle Tissue VEGF Level Analysis

Muscle VEGF expression was also assessed *ex vivo* on each of the imaging days [3–8] in a separate cohort of animals (*n*=4). Figures 3(c) and Table S4 show the % area expressing VEGF in the muscle from the ischemic and intact limbs on each of the days studied, and Figure 3(d) shows a representative field of view from ischemic muscle tissue on day 8. The % area expressing VEGF in the treated groups was comparable to the vehicle-treated ischemic leg on day 3 after surgical intervention. However, by day 8 the % area expressing VEGF was statistically increased in all treated groups (*p* < 0.05, 1-way ANOVA with post hoc Tukey's test). By day 14 and for the remaining time points, the % area expressing VEGF was only significantly higher in the combination-treated group (*p* < 0.05) compared to vehicle.

## 4. Discussion

The present study investigated the proangiogenic benefits of two well-known cardiovascular drugs for the treatment of PAD in a mouse model of diabetes using clinically relevant multimodal imaging techniques. Vasculature has been shown to rapidly remodel in response to ischemic insult [16], and nondiabetic mice have been shown to spontaneously regain much of their limb vascular volume within weeks of ligation [7]; diabetic animals, however, show much more limited vascular remodeling. Vascular repair is a biphasic process, with early revascularisation followed by vascular pruning [16], and proangiogenic therapies have been shown to alter these processes. PET imaging using [^18^F]FtRGD allows us to quantify the early angiogenic response to ischemia while routine clinical measures such as CT and MR angiography measure the resulting total vascular volume change [17–19]. Our data (Figures 2(a)–2(d)) show that diabetic animals display a significantly reduced spontaneous angiogenic response to ischemia and eventually a lower total vascular volume compared to nondiabetic animals, and this leads to significantly reduced functional recovery and increased disease progression and tissue necrosis.

The data in Figures 2(a) and 2(b) indicate that simvastatin, and to a lesser degree metformin, improve the initial angiogenic response after ischemic insult, improving early vascular remodeling similar to levels observed in nondiabetic control animals. When metformin and simvastatin were used in combination however, the early angiogenic response was synergistically improved beyond the levels observed in either therapy alone or in the nondiabetic controls, peaking at day 8. Immunohistochemical analysis of the ischemic muscle shows that this results from an increased expression of the proangiogenic protein VEGF, leading to an increased number of new CD31 positive blood vessels. New blood vessel formation occurs in all treated groups at the earlier time points, but in the combination-treated group, these effects are sustained. Vascular volume assessment with MR shows a significant improvement in the treated groups by day 21 with a much greater vascular volume in the combination-treated group compared to single treatment and nondiabetic controls and much greater than the vehicle-treated diabetic animals. These increases in angiogenesis and vascular volume lead to greater functional recovery of the affected limb and reduced progression of necrosis. While the current study uses a model of diabetes usually associated with type 1 diabetes, the majority of patients with PAD have type 2 diabetes. Further studies are needed to assess whether combined simvastatin and metformin treatment produces similar effects in a type 2 diabetes model where the effect of metformin may be more pronounced. Taken together, the data suggest that combined treatment with simvastatin and metformin enhances early hypoxia-dependent revascularisation, leading to greater vascular number and volume although the mechanism of action is unclear.

In diabetes, the development of peripheral artery disease is exacerbated by dysregulated glucose levels and increased oxidative stress. These processes disrupt the production of nitric oxide (NO), produced mainly by endothelial nitric oxide synthase (eNOS) [20] leading to slower and less complete revascularisation. Simvastatin has been shown to activate PI3K, increasing Akt phosphorylation by PDK-1 kinase and subsequently phosphorylation and activation of eNOS leading to angiogenesis and vasculogenesis [12]. Metformin not only reduces blood glucose levels, as shown in Figure 2(c), but also activates AMPK, which directly phosphorylates eNOS [9], so the effects of combined treatment could be due to synergistic effects on eNOS phosphorylation.

Both statins and metformin are routinely used clinically in diabetic patients and while they have been shown to have proangiogenic properties, they have not displayed the expected functional improvements in PAD patients when assessed retrospectively [21, 22]. This may be due to the fact that the prescribed simvastatin dose required for lipid altering effects is not optimal for angiogenesis, and statins are pleiotropic, displaying proangiogenic effects at lower doses and angiostatic effects at doses routinely used clinically for control of plasma lipid levels [23, 24].

## 5. Conclusions

In our present study, after combined treatment with simvastatin and metformin, we document a sustained improvement in limb angiogenesis, vascular volume, and functional recovery in a diabetic murine model of HLI. These results demonstrate the potential synergistic effect of these two therapies on revascularisation preclinically, and imaging with [^18^F]FtRGD potentially provides a robust method for early detection of these proangiogenic effects which may be useful for the assessment of proangiogenic therapies used to treat diabetic PAD patients.

## Figures and Tables

**Table 1:** Table of assessment criteria for limb damage and limb function after induction of hind limb ischemia.

Score	Limb damage	Functional use
1	No necrosis	Normal foot use for climbing and walking
2	Necrosis affecting one toenail	Reduced ability to grip when climbing/normal use when walking
3	Necrosis affecting two toenails or more	Foot not used for support when climbing/reduced use when walking
4	Necrosis affecting one toe	Foot not used for support when walking
5	Necrosis affecting two toes or more	Dragging limb

Impairment score was determined by multiplying the limb damage score by the functional use score (impairment score = limb damage score × functional use score).

## Data Availability

All supporting data are available through Singapore Bioimaging Consortium, Agency for Science, Technology and Research (A∗ STAR), 11 Biopolis Way, #07–10 Helios, Singapore 138667.

## Supplementary Materials

Supplementary Materials Supplementary materials includes detailed tables showing the [18F]FtRGD uptake (S1), MRI assessment of vascular volume (S2) and immunohistochemical assessment of CD31 (S3) and VEGF (S4) in both the ischemic and intact limbs of all groups studied.
