# Supplementary material for: Imaging the Proangiogenic Effects of Cardiovascular Drugs in a Diabetic Model of Limb Ischemia
Source: Contrast Media Mol Imaging. 2019 Feb 3;2019:2538909. doi: 10.1155/2019/2538909 (PMC6378011; doi:10.1155/2019/2538909)
Supplement: Supplementary Materials — includes detailed tables showing the [18F]FtRGD uptake (S1), MRI assessment of vascular volume (S2) and immunohistochemical assessment of CD31 (S3) and VEGF (S4) in both the ischemic and intact limbs of all groups studied. [file 2538909.f1.pdf]

Day	Vehicle Intact (%ID/g ± SD)	Vehicle Ischemic (%ID/g ± SD)	Metformin Intact (%ID/g ± SD)	Metformin Ischemic (%ID/g ± SD)	Simvastatin Intact (%ID/g ± SD)	Simvastatin Ischemic (%ID/g ± SD)	Combined Intact (%ID/g ± SD)	Combined Ischemic (%ID/g ± SD)	Non-diabetic Intact (%ID/g ± SD)	Non-diabetic Ischemic (%ID/g ± SD)
**1**	0.11 ± 0.04	0.63 ± 0.14	0.13 ± 0.06	0.60 ± 0.25	0.12 ± 0.05	0.54 ± 0.24	0.12 ± 0.06	0.50 ± 0.10	0.10 ± 0.03	0.52 ± 0.08
**3**	0.09 ± 0.03	0.58 ± 0.22	0.10 ± 0.03	0.68 ± 0.13	0.08 ± 0.04	0.62 ± 0.23	0.11 ± 0.04	0.65 ± 0.04	0.11 ± 0.02	0.66 ± 0.05
**8**	0.10 ± 0.02	0.62 ± 0.19	0.13 ± 0.04	1.01 ± 0.09	0.11 ± 0.03	1.16 ± 0.26	0.13 ± 0.03	1.28 ± 0.13	0.12 ± 0.04	1.09 ± 0.07
**14**	0.11 ± 0.01	0.59 ± 0.15	0.12 ± 0.04	0.67 ± 0.25	0.11 ± 0.03	0.87 ± 0.27	0.13 ± 0.09	0.98 ± 0.15	0.11 ± 0.05	0.91 ± 0.08
**21**	0.13 ± 0.01	0.50 ± 0.07	0.12 ± 0.03	0.49 ± 0.23	0.14 ± 0.03	0.61 ± 0.18	0.18 ± 0.10	0.84 ± 0.16	0.10 ± 0.02	0.61 ± 0.14
**28**	0.14 ± 0.05	0.48 ± 0.07	0.14 ± 0.04	0.44 ± 0.22	0.11 ± 0.09	0.48 ± 0.13	0.14 ± 0.05	0.52 ± 0.18	0.10 ± 0.03	0.42 ± 0.06

Table S1. Retention of $[^{18}F]$FtRGD measured by longitudinal PET imaging (~10 MBq, acquired from 70-90 mins post injection under isoflurane anaesthesia (data shown as %ID/g ± SD).

Day	Vehicle Intact (mm^3 ± SD)	Vehicle Ischemic (mm^3 ± SD)	Metformin Intact (mm^3 ± SD)	Metformin Ischemic (mm^3 ± SD)	Simvastatin Intact (mm^3 ± SD)	Simvastatin Ischemic (mm^3 ± SD)	Combined Intact (mm^3 ± SD)	Combined Ischemic (mm^3 ± SD)	Non-diabetic Intact (mm^3 ± SD)	Non-diabetic Ischemic (mm^3 ± SD)
**0**	5.36 ± 0.54	5.19 ± 0.61	5.14 ± 0.83	5.25 ± 0.53	5.31 ± 0.22	5.52 ± 0.18	5.01 ± 0.11	5.62 ± 0.29	5.22 ± 0.62	5.57 ± 0.54
**1**	5.22 ± 0.92	0.16 ± 0.06	5.05 ± 0.94	0.37 ± 0.41	5.17 ± 0.17	0.43 ± 0.36	5.24 ± 0.12	0.34 ± 0.10	5.31 ± 0.54	0.28 ± 0.11
**3**	5.09 ± 0.37	0.77 ± 0.93	4.76 ± 0.36	0.76 ± 0.16	5.23 ± 0.25	1.24 ± 0.68	4.93 ± 0.19	1.53 ± 0.57	5.19 ± 0.38	1.30 ± 0.78
**8**	5.24 ± 0.88	3.24 ± 0.76	5.39 ± 0.64	3.12 ± 0.03	5.18 ± 0.19	3.39 ± 0.72	4.92 ± 0.29	3.38 ± 0.35	5.23 ± 0.49	2.93 ± 0.57
**14**	5.47 ± 0.53	2.96 ± 0.77	5.69 ± 0.71	3.36 ± 1.70	5.00 ± 0.32	3.47 ± 0.64	4.83 ± 0.61	4.36 ± 0.36	5.29 ± 0.55	3.80 ± 0.56
**21**	4.86 ± 0.71	3.28 ± 0.24	5.48 ± 0.23	3.83 ± 0.74	5.08 ± 0.15	4.17 ± 1.06	5.11 ± 0.23	4.37 ± 0.19	5.38 ± 0.68	3.66 ± 0.57
**28**	5.17 ± 0.95	3.37 ± 0.38	5.32 ± 0.13	3.91 ± 0.52	5.20 ± 0.20	3.83 ± 0.61	5.13 ± 0.14	4.29 ± 0.11	5.17 ± 0.66	4.03 ± 0.64

Table S2. Hind limb vascular volume measured by TOF MRI (data shown as mean volume in mm^3 ± SD).

Day	Vehicle Intact (CD31 ± SD)	Vehicle Ischemic (CD31 ± SD)	Metformin Intact (CD31 ± SD)	Metformin Ischemic (CD31 ± SD)	Simvastatin Intact (CD31 ± SD)	Simvastatin Ischemic (CD31 ± SD)	Combined Intact (CD31 ± SD)	Combined Ischemic (CD31 ± SD)
**3**	30.7 ± 6.4	32.3 ± 11.7	33.0 ± 4.6	52.0 ± 8.9	37.3 ± 7.6	54.3 ±11.7	48.7 ± 9.0	56.0 ± 10.6
**8**	39.7 ± 10.1	68.3 ± 5.5	31.7 ± 9.5	81.3 ± 8.3	47.3 ± 3.1	87.7 ± 14.0	48.0 ± 13.1	108.7 ± 14.2
**14**	35.3 ± 2.5	50.3 ± 2.2	38.3 ± 10.0	65.0 ± 6.6	46.3 ± 11.2	88.4 ± 12.3	51.3 ± 5.8	101.7 ± 11.8
**21**	36.3 ± 5.1	43.3 ± 5.0	38.7 ± 4.7	66.0 ± 10.6	37.3 ± 14.6	78.0 ± 16.8	41.0 ± 3.6	94.0 ± 12.2
**28**	40.3 ± 3.2	46.3 ± 3.8	38.0 ± 11.5	65.2 ± 7.2	40.7 ± 6.7	74.2 ± 11.4	33.7 ± 10.2	88.7 ± 6.1

Table S3. Table showing the number of CD31 positive vessels in the hind limb muscle measured by immunohistochemical assessment of anti-CD-31 antibody staining (data shown as mean number of CD31 positively stained capillaries ± SD).

Day	Vehicle Intact (% VEGF ± SD)	Vehicle Ischemic (% VEGF ± SD)	Metformin Intact (% VEGF ± SD)	Metformin Ischemic (% VEGF ± SD)	Simvastatin Intact (% VEGF ± SD)	Simvastatin Ischemic (% VEGF ± SD)	Combined Intact (% VEGF ± SD)	Combined Ischemic (% VEGF ± SD)
**3**	9.7 ± 1.5	19.0 ± 2.6	10.7 ± 1.5	27.0 ± 5.6	9.3 ± 0.6	26.3 ± 4.5	9.3 ± 2.5	28.3 ± 2.5
**8**	9.0 ± 2.6	27.0 ± 10.4	13.0 ± 2.6	55.3 ± 4.2	11.7 ± 5.5	63.7 ± 16.8	11.0 ± 2.6	70.7 ± 12.1
**14**	10.0 ± 6.2	28.7 ± 3.1	14.3 ± 2.1	35.3 ± 10.3	7.3 ± 2.1	52.0 ± 12.5	12.0 ± 3.6	56.0 ± 9.2
**21**	11.7 ± 6.5	29.7 ± 11.9	13.3 ± 1.5	25.3 ± 3.1	11.3 ± 3.2	34.7 ± 6.5	13.7 ± 4.3	45.3 ± 5.5
**28**	9.7 ± 1.5	21.7 ± 7.1	11.0 ± 2.6	22.0 ± 4.0	10.0 ± 3.6	30.0 ± 6.6	10.7 ± 4.2	39.0 ± 6.1

Table S4. Table showing the percentage area of VEGF intense positive staining in hind limb muscle measured by immunohistochemical assessment of anti-VEGF antibody staining (data shown as % area positively stained for VEGF ± SD).
